# Beta-Blocker Use after Discharge in Patients with Acute Myocardial Infarction in the Contemporary Reperfusion Era

**DOI:** 10.3390/medicina58091177

**Published:** 2022-08-30

**Authors:** Mengjin Hu, Song Hu, Xiaojin Gao, Yuejin Yang

**Affiliations:** State Key Laboratory of Cardiovascular Disease, Fuwai Hospital, National Center for Cardiovascular Diseases, Chinese Academy of Medical Sciences & Peking Union Medical College, Beijing 100037, China

**Keywords:** drug therapy, beta-blockers, acute myocardial infarction, percutaneous coronary intervention, clinical outcomes

## Abstract

*Background and objectives:* The effect of beta-blocker use after discharge on patients with acute myocardial infarction (AMI) in the contemporary reperfusion era remains ambiguous. By applying meta-analysis, we sought to assess the role of beta-blockers in the contemporary reperfusion era. *Materials and Methods:* Randomized controlled trials (RCT) and observational studies using propensity score matching, comparing use of beta-blockers with non-use of beta-blockers, in patients with AMI after discharge. The primary outcome was all-cause mortality. Odds ratios (OR) and associated 95% confidence intervals (CI) were calculated. *Results*: One RCT and eight observational studies, containing 47,339 patients with AMI, were included. Compared with non-use of beta-blockers, beta-blocker use after discharge may have reduced the risk of all-cause mortality (OR: 0.70, 95% CI: 0.61 to 0.80, I^2^ = 14.4%), cardiac death (OR: 0.63, 95% CI: 0.44 to 0.91, I^2^ = 22.8%), myocardial infarction (OR: 0.73, 95% CI: 0.62 to 0.86, I^2^ = 0), and revascularization (OR: 0.92, 95% CI: 0.85 to 0.99, I^2^ = 0). No significant differences were found in major adverse cardiovascular events (MACE, OR: 0.88, 95% CI: 0.66 to 1.17, I^2^ = 78.4%), heart failure (OR: 0.56, 95% CI: 0.29 to 1.08, I^2^ = 0) or stroke (OR: 1.13, 95% CI: 0.92 to 1.39, I^2^ = 0). For patients with preserved left ventricular function, beta-blocker use after discharge may have also reduced the risk of all-cause mortality (OR: 0.61, 95% CI: 0.44 to 0.84, I^2^ = 0). *Conclusions*: Use of beta-blockers after discharge may still be beneficial for AMI patients in the contemporary reperfusion era, with or without preserved left ventricular function.

## 1. Introduction

Beta-blockers were among the first-line medications with improved clinical outcomes in patients with acute myocardial infarction (AMI), according to randomized controlled trials (RCT) conducted in the pre-reperfusion or thrombolytic era [1,2]. However, the last two decades have witnessed substantial evolution in the treatment of AMI, especially in the development and refinement of percutaneous coronary intervention (PCI), which has resulted in a significant decline in deaths [3]. Correspondingly, the American College of Cardiology (ACC)/American Heart Association (AHA) Guidelines, published in 2004, had already recommended PCI rather than fibrinolytic therapy for treatment of AMI (Class I, Level A) [4]. In this context, extrapolating the conclusions derived from the pre-perfusion or thrombolytic era to the contemporary PCI era may be not appropriate. However, RCTs investigating the effects of beta-blocker in the contemporary PCI era are limited. Observational studies were influenced by confounding factors, and showed conflicting results [5,6]. In previously published meta-analyses investigating the effect of beta-blocker use after discharge in the contemporary reperfusion era, only raw data from observational studies were recorded, without considering the impact of confounders [7,8,9]. To investigate the effect of beta-blocker use after discharge in the contemporary reperfusion era, and to minimize the effect of confounding factors, an updated systematic review and meta-analysis which only included RCTs and observational studies, with propensity score matching, was conducted. Moreover, the effect of beta-blocker use after discharge on patients with preserved left ventricular function was also assessed.

## 2. Methods

The present study was performed in compliance with the Preferred Reporting Items for Systematic Reviews and Meta-Analyses (PRISMA) 2020 statement [10], and was registered in the International Prospective Register of Systematic Reviews (CRD42022328398).

### 2.1. Study Selection

Two reviewers (M.J. Hu and S. Hu) independently searched PubMed, Web of Science, the Cochrane Library, ClinicalTrials.gov, and Google Scholar from publication to February 2022, using the following search terms: ‘acute coronary syndrome’; ‘ACS’; ‘myocardial infarction’; ‘MI’; ‘angiography’; ‘percutaneous coronary intervention’; ‘PCI’; ‘β-blockers’; and ‘beta-blockers’. References in the included articles and meta-analyses to similar topics were also carefully checked. Disagreements were resolved by discussing with the third-party investigator (X.J. Gao). Studies were selected according to the following criteria: RCTs or observational studies, with propensity score matching, that compared use of beta-blockers after discharge to non-use of beta-blockers after discharge, in patients with AMI, in the contemporary reperfusion era. Studies were excluded if they compared the clinical outcomes between different types or dosages of beta-blockers, or if the studies did not focus on patients with AMI.

### 2.2. Outcomes

The primary outcome was all-cause mortality. The secondary outcomes included major adverse cardiovascular events (MACE), cardiac death, myocardial infarction, heart failure, revascularization, and stroke. Clinical outcomes were recorded and defined according to per individual study. Information regarding study design, country, inclusion period, age, sex, the percentage of ST-segment elevation myocardial infarction (STEMI) and PCI, left ventricular ejection fractions (LVEF), prior heart failure, type of beta-blockers, clinical outcomes, and follow-up time were carefully extracted by the same two reviewers (M.J. Hu and S. Hu).

### 2.3. Statistical Analysis

The overall treatment effect was calculated under a random-effects model, expressed as odds ratios (OR) and 95% confidence intervals (CI). The level of heterogeneity was assessed using the I^2^-statistic test (I^2^ > 25%, >50%, >75% represented low, moderate, and high heterogeneity, respectively) [11]. The potential reason for heterogeneity was analyzed by meta-regression. A leave-one-out method was used to identify whether any individual study influenced the overall results. Publication bias was estimated according to the results of funnel plots or Begg’s test. *p* value < 0.05 represented statistical significance. All analyses were completed using STATA 16.0 (Stata Corp, College Station, TX, USA).

## 3. Results

### 3.1. Study Characteristics

**Figure 1:**
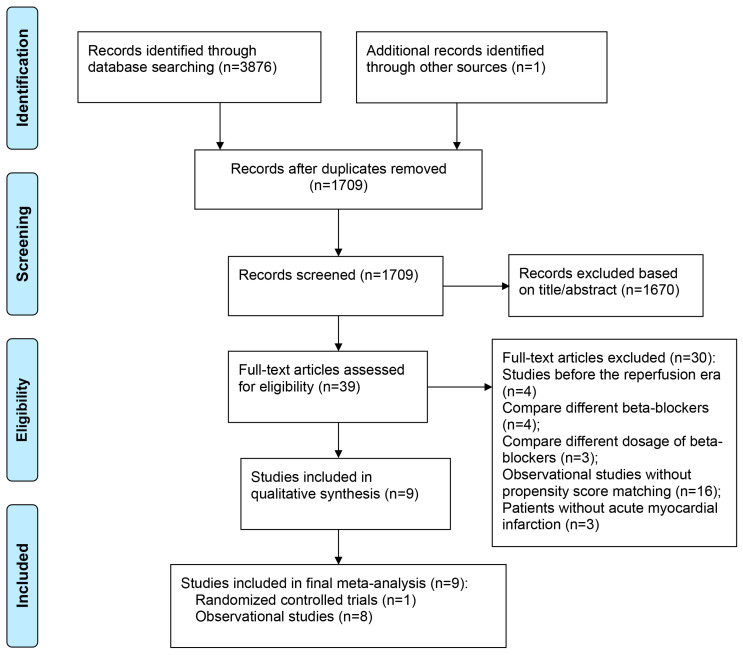
PRISMA Diagram for Study Inclusion.

Nine studies containing 47,339 patients (24,329 patients received beta-blockers, whereas 23,010 patients did not) were included in the present meta-analysis, including one RCT [12] and eight observational studies [13,14,15,16,17,18,19,20]. Details of the screening process for eligible studies are shown in Figure 1, and the baseline characteristics of the included studies and patients are presented in Table 1.

### 3.2. All-Cause Mortality

**Figure 2:**
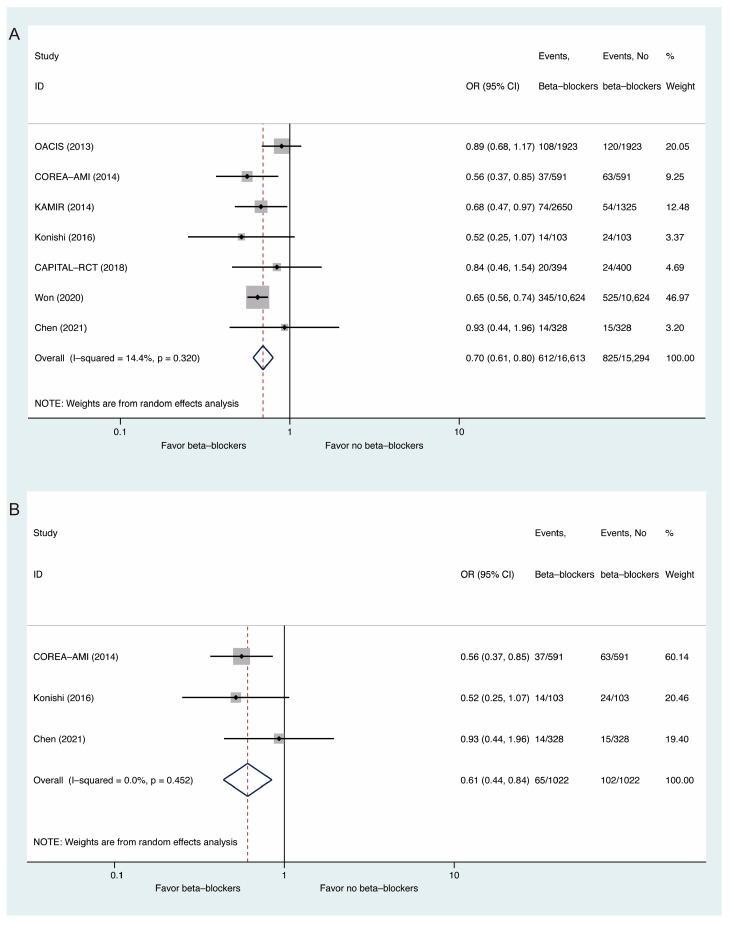
Comparisons of All-Cause Mortality Between Beta-Blockers and Controls: (**A**) whole populations; (**B**) populations with preserved left ventricular function.

Seven studies in total reported all-cause mortality. Compared with non-use of beta-blockers, beta-blocker use may have reduced the risk of all-cause mortality (OR: 0.70, 95% CI: 0.61 to 0.80, I^2^ = 14.4%, Figure 2A). For patients with preserved left ventricular function, a lower risk of all-cause mortality was also observed (OR: 0.61, 95% CI: 0.44 to 0.84, I^2^ = 0, Figure 2B). Meta-regression analysis of all-cause mortality revealed that age, sex, and the percentage of STEMI did not affect the relationship between beta-blocker use and all-cause mortality (Figure S1).

### 3.3. Secondary Outcomes

**Figure 3:**
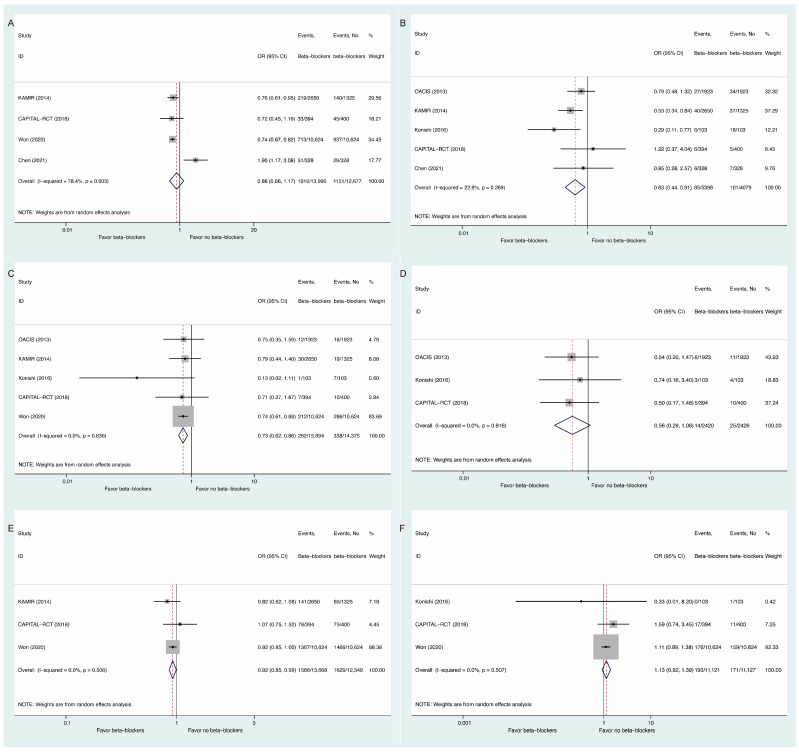
Comparisons of Secondary Outcomes Between Beta-Blockers and Controls: (**A**) major adverse cardiovascular events; (**B**) cardiac death; (**C**) myocardial infarction; (**D**) heart failure; (**E**) revascularization; (**F**) stroke.

For secondary outcomes, beta-blocker use may have decreased the risks of cardiac death (OR: 0.63, 95% CI: 0.44 to 0.91, I^2^ = 22.8%, Figure 3B), myocardial infarction (OR: 0.73, 95% CI: 0.62 to 0.86, I^2^ = 0, Figure 3C), and revascularization (OR: 0.92, 95% CI: 0.85 to 0.99, I^2^ = 0, Figure 3E), without significant influences on MACE (OR: 0.88, 95% CI: 0.66 to 1.17, I^2^ = 78.4%, Figure 3A), heart failure (OR: 0.56, 95% CI: 0.29 to 1.08, I^2^ = 0, Figure 3D) or stroke (OR: 1.13, 95% CI: 0.92 to 1.39, I^2^ = 0, Figure 3F).

### 3.4. Publication Bias and Sensitivity Analysis

The funnel plot (Figure S2) and Begg’s test (Figure S3) for primary and secondary outcomes revealed no publication bias. After performing leave-one-out analysis, consistent results were found (Figure S4).

## 4. Discussion

In this updated meta-analysis, including both RCT and observational studies with propensity score matching, we found that beta-blocker use after discharge may have reduced the risks of all-cause mortality, cardiac death, myocardial infarction, and revascularization without influence on MACE, heart failure or stroke. For patients with preserved left ventricular function, a lower risk of all-cause mortality was also found.

The evidence for beta-blocker use after AMI originates from the First International Study of Infarct Survival (ISIS-1) trial, published in 1986, where atenolol significantly reduced vascular death compared with non-use of beta-blockers (3.87% versus 4.57%, *p* < 0.05) [21]. However, in the Clopidogrel and Metoprolol in Myocardial Infarction Trial (COMMIT), published in 2005, the investigators found no significant differences in the co-primary endpoints of 30-day death, myocardial infarction, or cardiac arrest, between metoprolol and non-use of beta-blockers (*p* = 0.1) [22]. It is noteworthy that in the COMMIT trial, 54% of patients received fibrinolytic agents, 100% of patients received aspirin, and 50% of patients received dual antiplatelet therapy. However, in the ISIS-1 trial, only 5% of patients received antiplatelet agents. Deficiency in reperfusion and current medical treatment likely led to extensive myocardial scarring and subsequent fatal ventricular arrhythmias. Improved PCI techniques and increased use of aspirin, clopidogrel, and statins have substantially decreased all-cause mortality. Timely PCI can rescue more viable myocardium from necrosis, and prevent scar formation and left ventricular dysfunction, thereby further reducing the influence of beta-blockers [23]. As a result, it is hypothesized that the effectiveness of beta-blockers would be diminished or diluted by the modern treatment modality. The level of recommendation for use of beta-blockers has been downgraded in the recent ACC/AHA and European Society of Cardiology (ESC) Guidelines. The 2014 ACC/AHA Guidelines gave a Class IIa (Level C) recommendation in non-ST-segment elevation myocardial infarction patients with normal left ventricular function [24]. The 2020 ESC Guidelines also gave a Class IIa (Level B) recommendation in patients with prior myocardial infarction to reduce all-cause mortality, cardiovascular death, and cardiovascular morbidity [25].

Our results indicated that beta-blocker use after discharge may still reduce the risks of all-cause mortality, cardiac death, myocardial infarction, and revascularization in the contemporary reperfusion era. For patients with preserved left ventricular function, a lower risk of all-cause mortality was also found. Concordant with our results, the meta-analysis conducted by Maqsood, et al. [26], also suggested that beta-blocker treatment was associated with a reduced risk of all-cause mortality in patients with STEMI and preserved LVEF who underwent PCI. In the meta-analysis conducted by Dahl Aarvik and colleagues [7], beta-blocker treatment after discharge also reduced the risk of all-cause mortality [rate ratio (RR): 0.74, 95% CI: 0.64 to 0.85]. However, in the aforementioned meta-analyses, only raw data from observational studies were included, without considering the impact of confounders on the association. Therefore, in our meta-analysis, only RCTs and observational studies with propensity score matching were included, which enabled us to limit the influence of confounders as much as possible. Moreover, the overall heterogeneity was low in our meta-analysis, except for MACE, which may be explained by the different definitions of MACE.

The beneficial effects of beta-blockers in patients with AMI may be explained by multiple actions of beta-blockers on the heart. Firstly, monocyte recruitment to atherosclerotic plaques is significantly increased after AMI, resulting in the development of larger atherosclerotic lesions and more advanced morphology. Moreover, pain and anxiety alert the sympathetic nervous system, and activate neuroimmune synapses in the bone marrow, amplifying extramedullary myelopoiesis. However, these processes can be ameliorated by beta-blockers [27]. Secondly, adverse remodeling after AMI is associated with poor prognosis [28]. Beta-blockers have a beneficial effect on ventricular remodeling [29]. Thirdly, ventricular arrhythmia may increase the incidence of 90-day all-cause mortality [30]. Beta-blocker treatment could decrease the incidences of both short- and long-term ventricular arrhythmia [1,2]. Fourthly, oxygen supply to the affected portion of the heart is reduced in the context of AMI. The blockade of beta receptors results in slow heart rate, reduced myocardial contractility, and low systemic blood pressure, ultimately reducing myocardial workload and oxygen demand. 

While beta-blockers are relatively safe and inexpensive, they also have adverse effects [31]. For example, the incidence of coronary spasm was more common in patients receiving beta-blockers than in those receiving calcium antagonist (1.2% versus 0.2%, *p* = 0.02) [32], which requires our attention.

## 5. Limitations

Some limitations associated with the meta-analysis deserve attention. Firstly, only one RCT was included in our meta-analysis. The inclusion of observational studies may have biased our pooled estimates, because of the effect of confounding factors. We tried to limit this bias by only including data with propensity score matching. Secondly, statistical heterogeneity was high in MACE, which may be related to the different definitions of MACE. Thirdly, the types of beta-blockers were varied, and the dosages of beta-blockers were not available, which may have affected clinical outcomes. However, according to the randomized Carvedilol Acute Myocardial Infarction Study, different types of beta-blockers (carvedilol versus atenolol) had no effect on composite cardiovascular events (*p* = 0.99) [33]. In addition, there was no significant benefit from high-dose (≥25% of target dose) compared to low-dose (<25% of target dose) beta-blockers, for cardiac death [34].

## 6. Conclusions

Use of beta-blockers after discharge may still be beneficial for AMI patients in the contemporary reperfusion era, even for those with preserved left ventricular function.

## Figures and Tables

**Table 1:** Baseline Characteristics of Included Studies and Patients.

Study	Design	Country	Inclusion Period	Number of Patients	Age (years)	Male (%)	STEMI (%)	PCI (%)	LVEF (%)	Prior Heart Failure (%)	Beta-Blocker	Primary Outcomes	Follow-Up Time
OACIS [13], 2013	Observational	Japan	1998–2011	1923/1923	64.4/65.1	77.8/76.4	100/100	100/100	NA	NA	carvedilol (72.0%)	all-cause death	1430 days
COREA-AMI [14], 2014	Observational	Korea	2004–2009	591/591	62.9/63.0	69.5/69.5	57.5/57.2	100/100	60.7/60.2	NA	carvedilol (81.0%)	all-cause death	3 years
KAMIR [15], 2014	Observational	Korea	2005–2007	2650/1325	66/65	72.2/74.7	100/100	100/100	50/50	0.9/1.0	NA	all-cause death	367 days
Konishi [16], 2016	Observational	Japan	1997–2011	103/103	64.3/64.9	80.6/80.6	100/100	100/100	56.4/56.3	NA	NA	all-cause death	4.7 years
FAST-MI [17], 2016	Observational	France	2005	383/383	66.9/65.9	69/70	50/46	58/55	>50% (69.5/69.5)	0	Acebutolol (22%), Atenolol (33%), Bisoprolol (29%), Metoprolol (7%)	all-cause death	5 years
CAPITAL-RCT [12], 2018	Randomized controlled trial	Japan	2010–2014	394/400	63.9/64.5	83/78	100/100	100/100	≥40%	0	carvedilol	all-cause death, MI, hospitalization for heart failure, and hospitalization for acute coronary syndrome	3.9 years
Lee [18], 2020	Observational	Korea	2013–2017	7333/7333	65/65	75.2/75.2	AMI	100/100	NA	3.9/4.0	Carvedilol (45.7), Bisoprolol (29.6), Nebivolol (8.3)	all-cause death	2.2 years
Won [19], 2020	Observational	Korea	2005–2014	10,624/10,624	62/61	75.19/75.22	AMI	100/100	NA	4.75/4.64	NA	all-cause death, MI, and stroke	2 years
Chen [20], 2021	Observational	China	2010–2017	328/328	58.7/ 60.0	83.8/81.7	93.3/90.9	100/100	57.6/58.0	0	Atenolol, Bisoprolol, Metoprolol	all-cause death	1 year

Beta-blockers/No beta-blockers; ACS: acute coronary syndrome; AMI: acute myocardial infarction; LVEF: left ventricular ejection fraction; MACE: major adverse cardiac events; MI: myocardial infarction; NA: not available; PCI: percutaneous coronary intervention; STEMI: ST-segment elevation myocardial infarction; TVR: target vessel revascularization.

## Data Availability

The data presented in this study are available on request from the corresponding author.

## Supplementary Materials

The following supporting information can be downloaded at: https://www.mdpi.com/article/10.3390/medicina58091177/s1, Figure S1: Meta-regression for All-Cause Mortality; Figure S2: Funnel Plot of Publication Bias for Primary and Secondary Outcomes; Figure S3: Begg’s Test for Primary and Secondary Outcomes; Figure S4: Leave-One-Out Analyses for Primary and Secondary Outcomes.
