# Supplementary material for: Beta-Blocker Use after Discharge in Patients with Acute Myocardial Infarction in the Contemporary Reperfusion Era
Source: Medicina (Kaunas). 2022 Aug 30;58(9):1177. doi: 10.3390/medicina58091177 (PMC9506114; doi:10.3390/medicina58091177)
Supplement: Supplementary file 1 [file medicina-58-01177-s001.zip › Supplementary Figure.pdf]

Supplementary Figure S1 Meta-regression for All-Cause Mortality

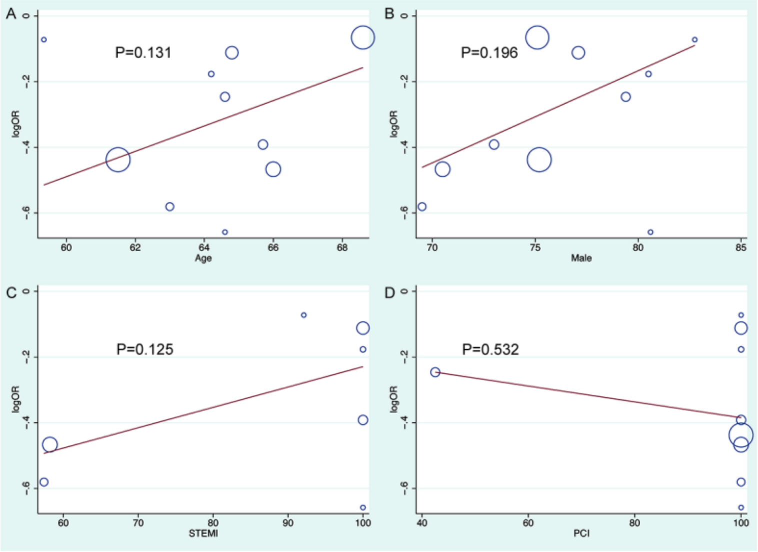

A: age; B: male; C: STEMI

STEMI: ST-segment elevation myocardial infarction

Supplementary Figure S2 Funnel Plot of Publication Bias for Primary and Secondary Outc

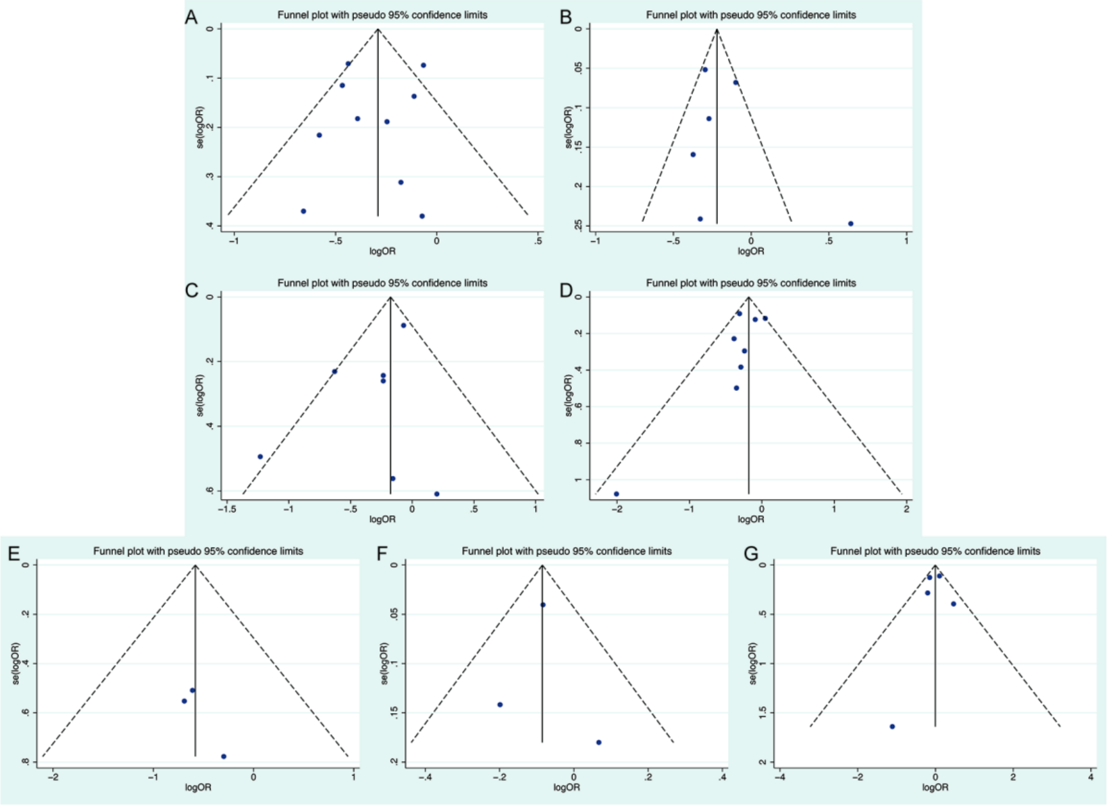

omes

A: all-cause mortality; B: major adverse cardiovascular events; C: cardiac death; D: myocardial infarction; E: heart failure; F: revascularization; G: stroke

Supplementary Figure S3 Begg's Test for Primary and Secondary Outcomes

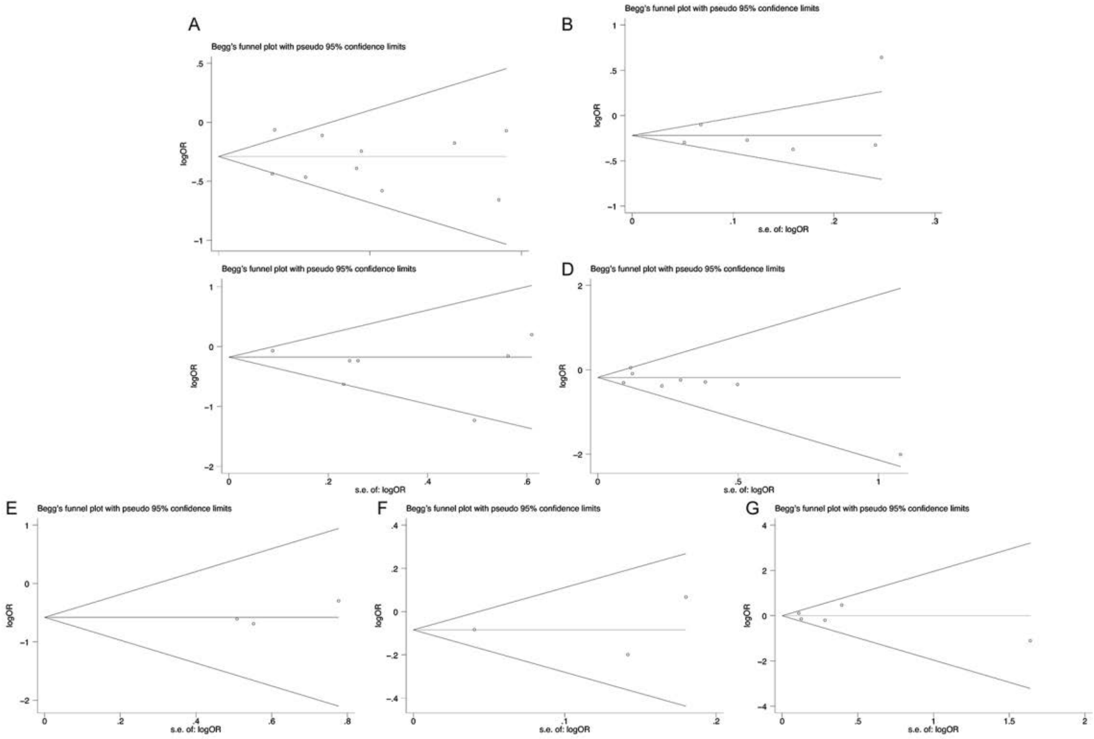

A: all-cause mortality; B: major adverse cardiovascular events; C: cardiac death; D: myocardial infarction; E: heart failure; F: revascularization; G: stroke

Supplementary Figure S4 Leave-One-Out Analyses for Primary and Secondary Outcomes

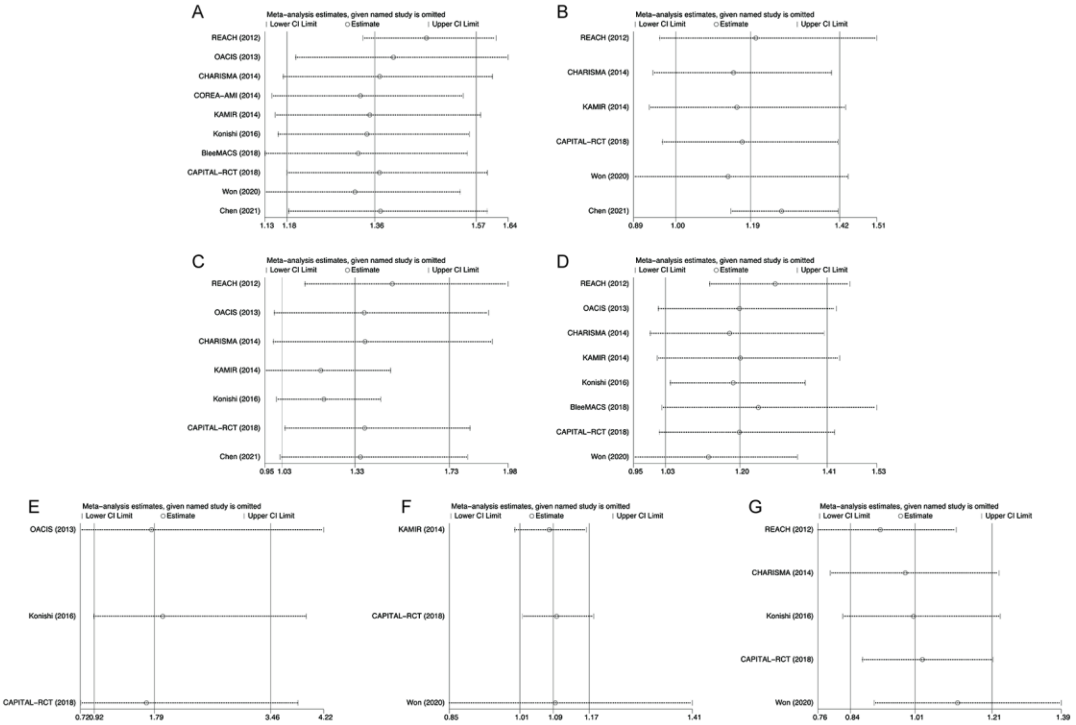

A: major adverse cardiovascular events; B: cardiac death; C: myocardial infarction; D: heart failure; E: revascularization; F: stroke
